# Notch signaling drives development of Barrett’s metaplasia from Dclk1-positive epithelial tuft cells in the murine gastric mucosa

**DOI:** 10.1038/s41598-021-84011-4

**Published:** 2021-02-24

**Authors:** Bettina Kunze, Moritz Middelhoff, H. Carlo Maurer, Tatiana Agibalova, Akanksha Anand, Anne-Marie Bührer, Hsin-Yu Fang, Theresa Baumeister, Katja Steiger, Julia Strangmann, Roland M. Schmid, Timothy C. Wang, Michael Quante

**Affiliations:** 1 Klinik und Poliklinik für Innere Medizin II, Technical University of Munich, Munich, Germany; 2 Institute of Pathology, Technical University of Munich, Munich, Germany; 3 Department of Medicine, Columbia University Medical Center, New York, NY USA; 4 Klinik für Innere Medizin II, Gastrointestinale Onkologie, Universitätsklinikum Freiburg, Freiburg, Germany

**Keywords:** Cancer, Cancer microenvironment, Gastrointestinal cancer, Barrett oesophagus

## Abstract

Barrett’s esophagus (BE) is a precursor to esophageal adenocarcinoma (EAC), but its cellular origin and mechanism of neoplastic progression remain unresolved. Notch signaling, which plays a key role in regulating intestinal stem cell maintenance, has been implicated in a number of cancers. The kinase Dclk1 labels epithelial post-mitotic tuft cells at the squamo-columnar junction (SCJ), and has also been proposed to contribute to epithelial tumor growth. Here, we find that genetic activation of intracellular Notch signaling in epithelial Dclk1-positive tuft cells resulted in the accelerated development of metaplasia and dysplasia in a mouse model of BE (pL2.Dclk1.N2IC mice). In contrast, genetic ablation of Notch receptor 2 in Dclk1-positive cells delayed BE progression (pL2.Dclk1.N2fl mice), and led to increased secretory cell differentiation. The accelerated BE progression in pL2.Dclk1.N2IC mice correlated with changes to the transcriptomic landscape, most notably for the activation of oncogenic, proliferative pathways in BE tissues, in contrast to upregulated Wnt signalling in pL2.Dclk1.N2fl mice. Collectively, our data show that Notch activation in Dclk1-positive tuft cells in the gastric cardia can contribute to BE development.

## Introduction

Barrett´s esophagus (BE) is characterized by the replacement of the normal esophageal squamous mucosa by a metaplastic columnar gastric mucosa, typically following damage by gastroesophageal reflux disease (GERD). Both BE and GERD are known major risk factors for the development of esophageal adenocarcinoma (EAC)^1^. To study BE formation and progression to EAC in vivo, we established a mouse model expressing human IL-1beta cDNA downstream of an Epstein-Barr-Virus promoter (ED-L2), which targets the oral cavity, esophagus and squamous forestomach of the mouse^2,3^. pL2-IL1b mice robustly develop by 6 months of age chronic inflammation and epithelial hyperplasia at the squamo-columnar junction (SCJ), which progresses to severe columnar metaplasia by 12–15 months of age and then to high-grade dysplasia (HGD) or EAC formation at later timepoints, thus mimicking human BE pathology^3^.

Notch signaling has been implicated in the regulation of intestinal stem cell activity. Notch inactivation through genetic or pharmacologic manipulation has been shown to halt epithelial proliferation while inducing predominant secretory cell differentiation^4^, whereas spatial Notch activation appears to favor dedifferentiation and confer features of stemness in the gastric epithelium^5^. Accordingly, active Notch signaling appears to promote intestinal tumor formation^6^, and we reported that Dll1-mediated activation of Notch signaling contributes to development of dysplasia at the SCJ in a BE mouse model^3^. However, the cell of origin for BE development remains unsettled, with squamous cells^7^, transitional basal cells^8^, squamous duct gland cells^9^, and gastric cardia cell origins^10^ all proposed as possible candidates.

The microtubule-associated kinase DCLK1 was originally proposed to identify adult gastric^11^ and colonic progenitor cells^12^, but more recently numerous studies have provided evidence that DCLK1 predominantly labels differentiated gastrointestinal tuft cells. These communicate with the immune system^13–16^, contribute to neuronal signaling in the intestinal stem cell niche^17^ and provide additional important niche signals to the epithelium^18^. Interestingly, Dclk1-positive tuft cells are abundant in the gastric cardia, while they are present at much lower numbers in the distal stomach, small intestine and colon^19^. In intestinal metaplastic tissue, Dclk1-positive epithelial cells expand^20,21^, and the number of Dclk1-postive cells also increases in metaplastic BE lesions^3^. While progression to high-grade dysplasia or cancer is associated with a decline in Dclk1-positive epithelial cells^20^, their expansion during metaplasia suggests a possible role during early tumorigenesis. Indeed, the combination of inflammatory injury and an oncogenic mutation are able to induce colonic tumor formation from Dclk1-positive epithelial cells^22^, and the kinase Dclk1 itself has been implicated in the promotion of intestinal tumor growth^23^.

Thus, we here investigated the role of Notch signaling in Dclk1-positive gastric epithelial tuft cells during BE development and progression, which was modelled by pL2-IL1b mice^3^. To genetically modify Dclk1-positive gastric tuft cells we employed transgenic Dclk1-CreERT2 mice^22^ crossed with transgenic mice carrying either a loxP-flanked Notch-2IC knock-in allele^24^ or loxP sites in the N2 locus^25^. Tamoxifen-mediated induction of Cre activity then resulted in increased intra-cellular Notch-2IC signaling or N2 inactivation, which decreased intracellular Notch signaling in Dclk1-positive cells. Interestingly, using pL2-IL1b;Dclk1-CreERT2;N2IC^F/F^ mice resulted in a marked acceleration of the BE phenotype of pL2-IL1b mice. This was evident by the presence of increased dysplastic lesions and crypt fission events, and correlated with a marked decrease in overall survival. In contrast, genetic ablation of the Notch 2 receptor in Dclk1-positive cells (pL2-IL1b;Dclk1-CreERT2;N2^F/F^ mice) resulted in a milder BE phenotype compared to controls, accompanied by increased secretory differentiation in metaplastic lesions. Overall, this data demonstrates that columnar Dclk1-positive epithelial tuft cells can modulate BE development and progression in a Notch-dependent manner.

## Results

### Notch activation in Dclk1-positive gastric tuft cells accelerates BE progression

First, we established that pL2-IL1b; Dclk1-CreERT2 mice (or pL2.Dclk1 mice) developed BE lesions compared to control WT mice (genotype negative littermates), as similar to our previous reports for the pL2-IL1b mice^3^. To induce transgenic pL2.Dclk1 and subsequent experimental mice we employed three administrations of tamoxifen (6 mg each) within a week at 6 months of age (Supplemental Fig. 1A). This model conferred the advantage of the temporal acceleration of the phenotype as it has been described to induce a transient gastric metaplasia^26^, therefore all subsequent experimental and control groups were induced similarly. In this experimental setup, pL2.Dclk1 mice developed metaplasia and dysplasia concomitant with acute inflammation at the indicated timepoints, while WT mice did not exhibit any phenotypic alterations (Supplemental Fig. 1B–D). This also translated into reduced long-term survival and weight of pL2.Dclk1 mice (Supplemental Fig. 1E,F). In line with the accelerated onset of metaplasia by tamoxifen and the described expansion of Dclk1-positive tuft cells in metaplastic tissues^3^, we observed an expansion of gastric DCLK1-positive tuft cells in induced pL2.Dclk1 tissues (Supplemental Fig. 2A,B). However, tamoxifen administration did not appear to change epithelial Notch activation (Supplemental Fig. 2C,D).

**Figure 1:**
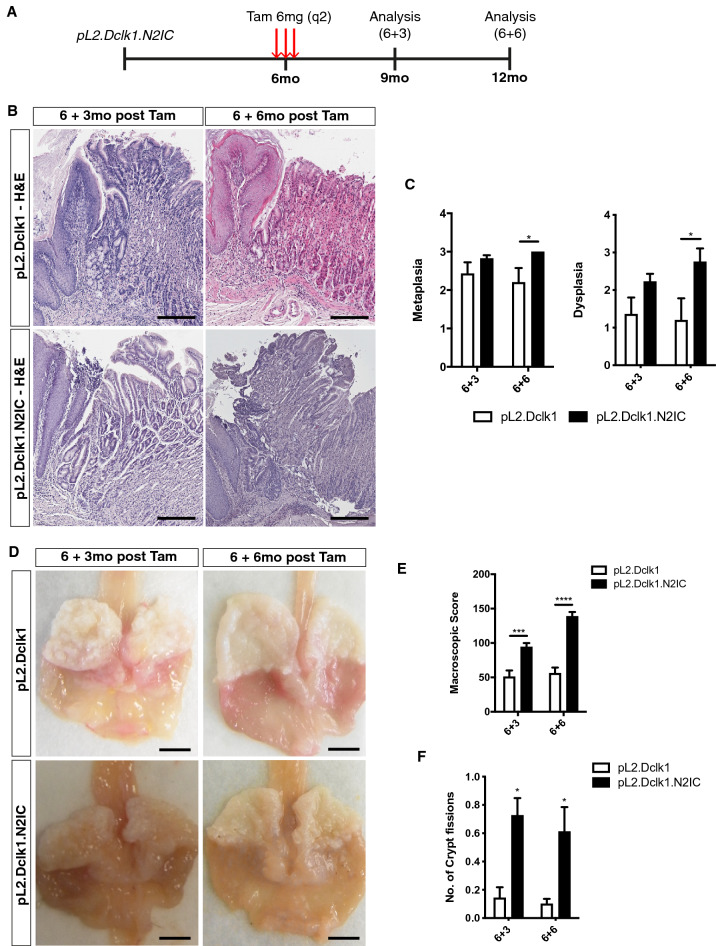
Notch activation in Dclk1-positive gastric tuft cells accelerates BE progression. (**A**) Experimental scheme illustrating timepoints of tamoxifen induction and tissue collection for analysis. (**B**) Representative H&E pictures of the SCJ of pL2.Dclk1 and pL2.Dclk1.N2IC mice, respectively; scale bars = 200 µm. (**C**) Histopathologic scoring in pL2.Dclk1.N2IC compared to pL2.Dclk1 controls for metaplasia (6 + 3 n = 23 pL2.Dclk1.N2IC, n = 7 pL2.Dclk1 controls; ordinary two-way ANOVA, p = 0.029) and dysplasia (6 + 3 n = 20 pL2.Dclk1.N2IC, n = 7 pL2.Dclk1 controls; ordinary two-way ANOVA, p = 0.033). (**D**) Representative macroscopic pictures of the opened stomach of pL2.Dclk1 and pL2.Dclk1.N2IC mice, respectively; scale bars = 0.5 cm. (**E**) Macroscopic scoring in pL2.Dclk1.N2IC compared to pL2.Dclk1 controls (6 + 3 n = 14 pL2.Dclk1, n = 42 pL2.Dclk1.N2IC mice, ordinary two-way ANOVA, p = 0.0001; 6 + 6 n = 8 pL2.Dclk1, n = 22 pL2.Dclk1.N2IC mice, ordinary two-way ANOVA, p ≤ 0.0001). (**F**) Histopathologic scoring in pL2.Dclk1.N2IC mice compared to pL2.Dclk1 controls for the number of crypt fissions (6 + 3 n = 7 pL2.Dclk1, n = 19 pL2.Dclk1.N2IC mice, multiple t test, adj. p = 0.019; 6 + 6 n = 6 pL2.Dclk1, n = 9 pL2.Dclk1.N2IC mice, multiple t test, adj. p = 0.035); error bars indicate SEM.

In a previous study, we showed that Notch 2 was highly upregulated in comparison to Notch 1 in the cardia of pL2-IL1b mice^10^. Furthermore, analysis of single-cell data on intestinal tuft cells^27^ confirmed the expression of Notch pathway members in tuft cells, indicating that gastric tuft cells may contribute to BE pathogenesis. To study the effect of Notch 2 overexpression in Dclk1-positive gastric tuft cells on BE progression, we generated pL2-IL1b;Dclk1-CreERT2;N2IC^F/F^ mice (or pL2.Dclk1.N2IC mice) and analyzed the mice at 3 and 6 months post induction (6 + 3 and 6 + 6, respectively) (Fig. 1A). Indeed, N2IC overexpression in Dclk1-positive tuft cells prominently accelerated BE progression, similar to Notch activation in Lgr5-positive gastric progenitor cells^10^. Histologic analysis confirmed the accelerated formation of metaplasia and dysplasia (Fig. 1B,C), and macroscopic scoring revealed a significant alteration in macroscopic lesions of the gastric cardia (Fig. 1D,E). The accelerated BE phenotype was associated with significantly upregulated crypt fission, which has been associated with increased Notch signaling^28^ (Fig. 1F).

### Metaplastic lesions in pL2.Dclk1.N2IC mice are partially derived from Dclk1-positive gastric tuft cells

**Figure 2:**
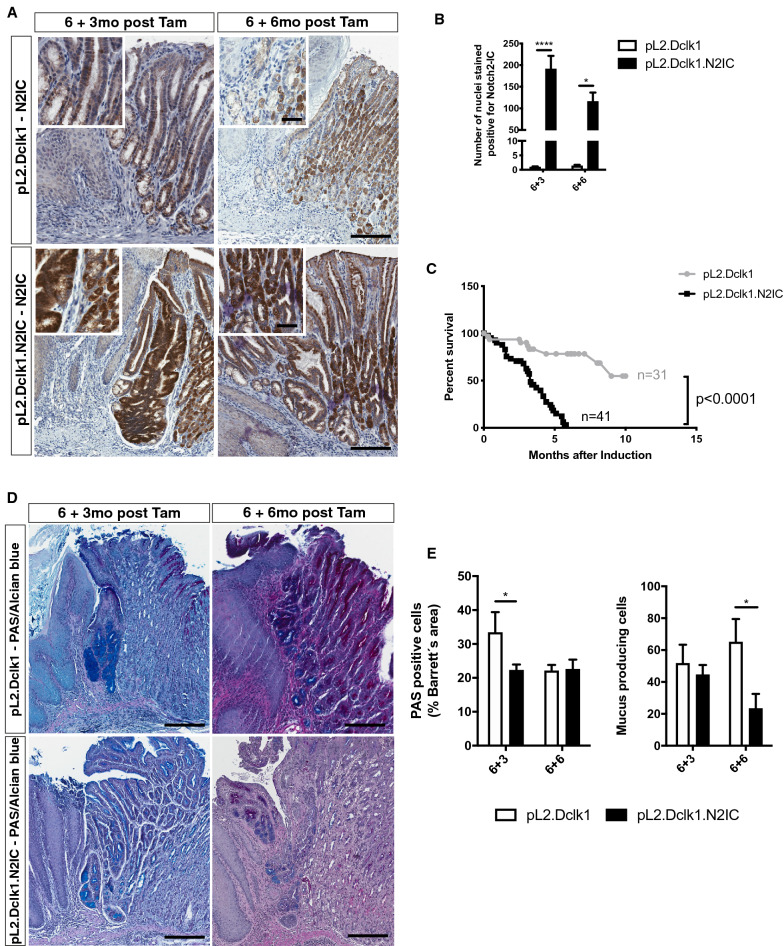
Metaplastic lesions in pL2.Dclk1.N2IC mice are partially derived from Dclk1-positive gastric tuft cells. (**A**) Representative stainings for Notch2-IC of the SCJ of pL2.Dclk1 and pL2.Dclk1.N2IC mice, respectively; scale bars = 200 µm; insets scale bar = 50 µm. (**B**) Histopathologic scoring of Notch2-IC stained nuclei in pL2.Dclk1.N2IC tissues compared to pL2.Dclk1 controls (6 + 3 n = 25 pL2.Dclk1.N2IC, n = 11 pL2.Dclk1 controls; ordinary two-way ANOVA, p ≤ 0.0001; 6 + 6 n = 20 pL2.Dclk1.N2IC, n = 7 pL2.Dclk1 controls; ordinary two-way ANOVA, p = 0.046). (**C**) Survival analysis of pL2.Dclk1.N2IC compared to pL2.Dclk1 controls shows significant decrease of median overall survival in pL2.Dclk1.N2IC mice (n = 41 pL2.Dclk1.N2IC; n = 31 pL2.Dclk1 controls; Median survival pL2.Dclk1.N2IC = 3.3 months; Median survival pL2.Dclk1 ≥ 10 months; log-rank (Mantel-Cox) test, Chi square = 25,91, p ≤ 0.0001). (**D**, **E**) Histopathologic analysis and scoring in pL2.Dclk1.N2IC tissues compared to pL2.Dclk1 controls for PAS-positive cells at the SCJ (6 + 3 n = 21 pL2.Dclk1.N2IC, n = 6 pL2.Dclk1 controls; ordinary two-way ANOVA, p = 0.025; 6 + 6 n = 9 pL2.Dclk1.N2IC, n = 2 pL2.Dclk1 controls) and mucous producing cells (6 + 3 n = 21 pL2.Dclk1.N2IC, n = 6 pL2.Dclk1 controls; 6 + 6 n = 6 pL2.Dclk1.N2IC, n = 4 pL2.Dclk1 controls, ordinary two-way ANOVA, p = 0.049); scale bars = 200 µm; error bars indicate SEM.

To investigate the effect of genetic activation of Notch signaling in Dclk1-positive gastric tuft cells, we first confirmed by immunohistochemistry for Notch2-IC a significant increase in positively stained nuclei in induced pL2.Dclk1.N2IC mice compared to controls (Fig. 2A,B). In addition, *Notch2* mRNA expression showed significant upregulation in induced cardia tissue (Supplemental Fig. 3A). To address the effects of Notch activation on Dclk1-positive tuft cell activity, we evaluated recombination of the reporter cassette R26-LacZ in control mice and pL2.Dclk1 and the pL2.Dclk1.N2IC mice, respectively. In Dclk1-CreERT2; R26-LacZ control mice, we did not observe tracing of cardia glands following induction (Supplemental Fig. 3B). However, induced pL2.Dclk1.LacZ mice showed the existence of single clonally labeled crypts at the SCJ at 3 months after reporter induction, indicating that chronic inflammation alone may enable dedifferentiation from Dclk1-positive tuft cells (6 + 3; Supplemental Fig. 3C). The lacZ-positive cells appeared restricted to the epithelial cell compartment and limited to cardia crypts close to the SCJ. In line with the increase in metaplasia and dysplasia, genetic Notch activation in Dclk1-positive cells resulted in more prominent tracing from Dclk1-positive tuft cells (Supplemental Fig. 3C). In comparison to pL2.Dclk1 control tissues, some Dclk1-positive tuft cells in the cardia of pL2.Dclk1.N2IC mice showed positive overlap with nuclear Ki67 (Supplemental Fig. 3D), thus corroborating their potential dedifferentiation in this model. Lastly, we could not detect positive overlap of DCLK1 with Lgr5-EGFP in the cardia (Supplemental Fig. 3E), however this does not exclude that during metaplasia Lgr5-positive gastric stem cells may contribute to the observed phenotype by upregulating *Dclk1* expression.

In line with the accelerated BE phenotype of pL2.Dclk1.N2IC mice, overall survival was drastically decreased in these mice (Fig. 2C), preceded by profound weight loss (Supplemental Fig. 3F). Thus, these findings suggest that Notch is capable of activating Dclk1-positive gastric cardia tuft cells, similar to previously described changes in Notch-activated parietal cells of the corpus^5^. In line with Notch promoting BE progression, mucus producing cells in BE lesions of pL2.Dclk1.N2IC mice were significantly reduced (Fig. 2D,E). Finally, since chronic inflammation is a known driver of BE^29^, we sought to investigate whether the accelerated BE phenotype of pL2.Dclk1.N2IC mice was associated with an increased presence of stromal immune cells. However, histopathologic scoring of inflammation at the SCJ of pL2.Dclk1.N2IC mice did not reveal significant changes (Supplemental Fig. 3G), and analysis of immune cell populations from the cardia region of pL2.Dclk1 vs. pL2.Dclk1.N2IC mice showed no significant differences (not shown), similar to our recent data of pL2.Lgr5 tissues^10^. Thus, these data suggest that Notch activation in pL2.Dclk1.N2IC mice promotes epithelial-intrinsic signaling and expansion from Dclk1-positive cells^30^.

### Genetic ablation of the Notch 2 receptor in Dclk1-positive tuft cells decelerates BE progression

**Figure 3:**
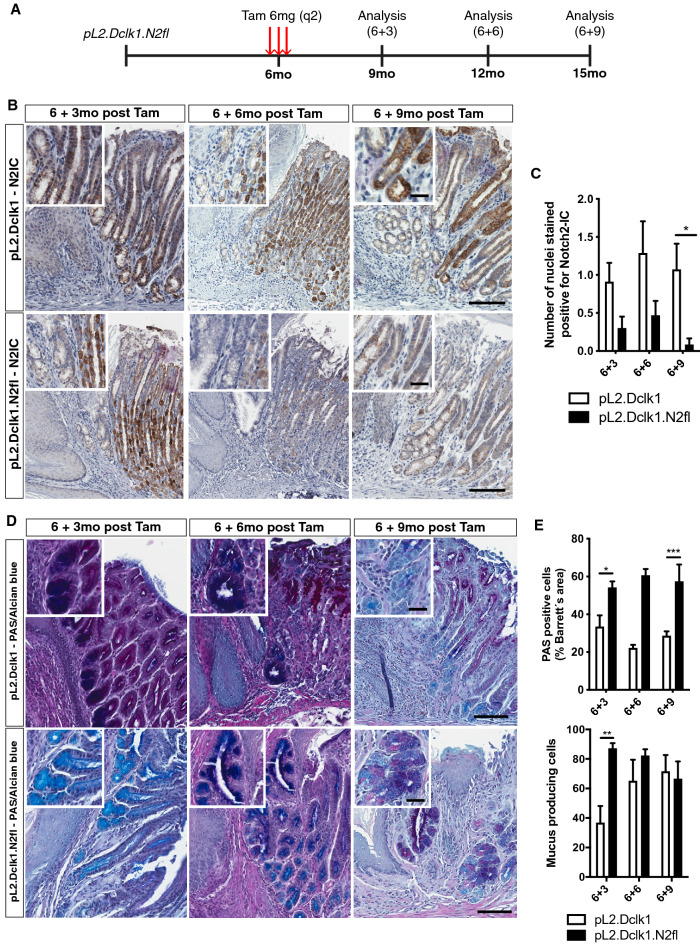
Genetic ablation of the Notch 2 receptor in Dclk1-positive gastric tuft cells decelerates BE progression. (**A**) Experimental scheme illustrating timepoints of Tamoxifen induction and tissue collection for analysis. (**B**) Representative stainings for Notch2-IC of the SCJ of pL2.Dclk1 and pL2.Dclk1.N2fl mice, respectively; scale bars = 200 µm; insets scale bar = 50 µm; for pL2.Dclk1, at 6 + 3 and 6 + 6 the same pictures are shown as in Fig. 2A for comparison to pL2.Dclk1.N2fl tissues. (**C**) Histopathologic scoring of Notch2-IC stained nuclei in pL2.Dclk1.N2fl tissues compared to pL2.Dclk1 controls (6 + 3 n = 10 pL2.Dclk1.N2fl, n = 11 pL2.Dclk1 controls; 6 + 6 n = 15 pL2.Dclk1.N2fl, n = 7 pL2.Dclk1 controls; 6 + 9 n = 12 pL2.Dclk1.N2fl, n = 14 pL2.Dclk1 controls, ordinary two-way ANOVA, p = 0.014). (**D**, **E**) Histopathologic analysis and scoring in pL2.Dclk1.N2fl tissues compared to pL2.Dclk1 controls for PAS-positive cells at the SCJ (6 + 3 n = 6 pL2.Dclk1.N2fl, n = 6 pL2.Dclk1 controls, ordinary two-way ANOVA, p = 0.017; 6 + 6 n = 9 pL2.Dclk1.N2fl, n = 2 pL2.Dclk1 controls; 6 + 9 n = 5 pL2.Dclk1.N2fl, n = 7 pL2.Dclk1 controls, ordinary two-way ANOVA, p = 0.0009) and mucous producing cells (6 + 3 n = 5 pL2.Dclk1.N2fl, n = 6 pL2.Dclk1 controls, ordinary two-way ANOVA, p = 0.0057; 6 + 6 n = 9 pL2.Dclk1.N2fl, n = 4 pL2.Dclk1 controls; 6 + 9 n = 7 pL2.Dclk1.N2fl, n = 7 pL2.Dclk1 controls); scale bars = 200 µm; insets scale bar = 50 µm; error bars indicate SEM.

Given the finding in the pL2.Dclk1.N2IC model that Notch-activated Dclk1-positive tuft cells appeared to be important contributors to BE formation, we wondered whether Notch blockade in these cells attenuates the BE phenotype and thus confirms their regulatory role in BE. Thus, genetic ablation of the Notch 2 receptor was achieved by creating pL2.Dclk1.N2fl mice, which received repeated doses of Tam at 6 months of age (Fig. 3A). Immunohistochemical staining for Notch2-IC in (cardia) tissues from these mice indeed confirmed a gradual decrease in Notch2-IC expression over time following tamoxifen induction (Fig. 3B,C). This translated into a significantly reduced macroscopic phenotype at later timepoints (Supplemental Fig. 4A, B). Interestingly, we could also observe a prominent decrease of DCLK1-positive tuft cells in pL2.Dclk1.N2fl tissues (Supplemental Fig. 4C). Also, we observed a trend towards reduced dysplasia (Supplemental Fig. 4D), which coincided with an increase of mucus-secreting cells, thus revealing a shift from dysplasia to metaplasia (Fig. 3D,E). Furthermore, overall survival did substantially differ from pL2.Dclk1.N2IC mice, in that we observed long term survival (up to 6 + 9 timepoint of analysis) and a positive survival trend in pL2.Dclk1.N2fl vs. pL2.Dclk1 mice (Supplemental Fig. 4E). Taken together, this data suggests that Dclk1-positive epithelial cells of the cardia contribute to BE progression in part through Notch signaling, which can be partially abrogated by genetic ablation of the Notch 2 receptor.

### Notch signaling confers epithelial-intrinsic modulations of organoid growth and differentiation

**Figure 4:**
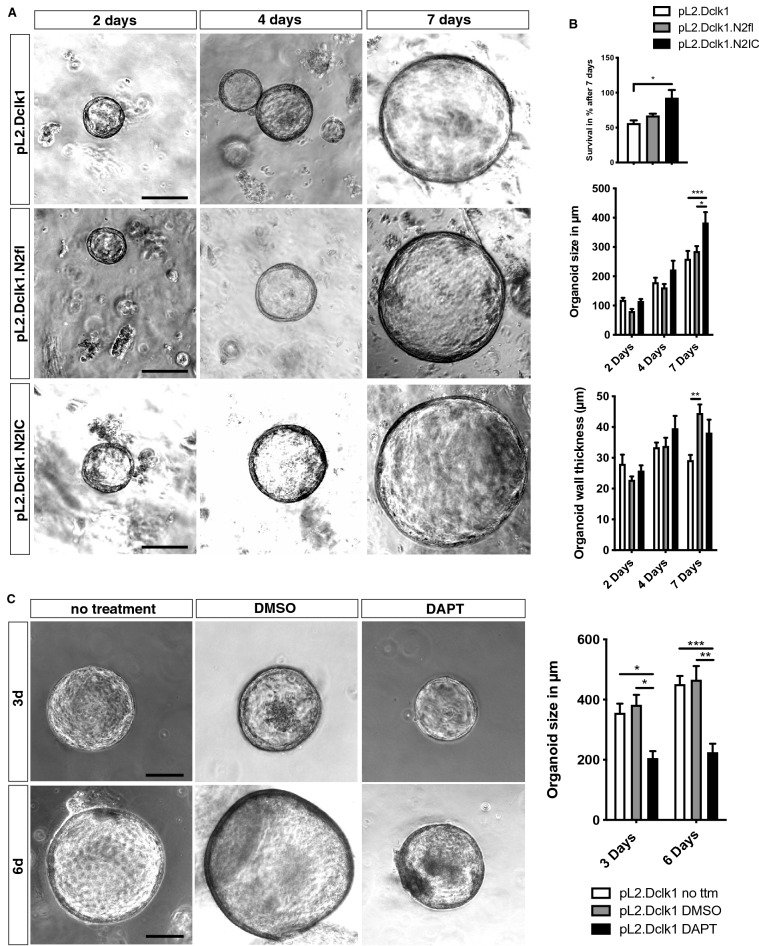
Notch signaling confers epithelial-intrinsic modulations of organoid growth and differentiation. (**A**) Representative brightfield pictures of cardia organoids of pL2.Dclk1, pL2.Dclk1.N2fl and pL2.Dclk1.N2IC mice, respectively; scale bars = 100 µm. (**B**) Organoid survival and size showed significantly increased in organoids from pL2.Dclk1.N2IC mice (measurements n = 6 pL2.Dclk1, n = 5 pL2.Dclk1.N2fl, n = 4 pL2.Dclk1.N2IC, Kruskal–Wallis test, pL2.Dclk1 vs. pL2.Dclk1.N2IC p = 0.028), organoid size (2d, measurements n = 47 pL2.Dclk1, n = 15 pL2.Dclk1.N2fl, n = 19 pL2.Dclk1.N2IC; 4d n = 39 pL2.Dclk1, n = 53 pL2.Dclk1.N2fl, n = 12 pL2.Dclk1.N2IC; 7d n = 29 pL2.Dclk1, n = 20 pL2.Dclk1.N2fl, n = 17 pL2.Dclk1.N2IC; ordinary two-way ANOVA, pL2.Dclk1 vs. pL2.Dclk1.N2IC p = 0.001, pL2.Dclk1.N2fl vs. pL2.Dclk1.N2IC p = 0.007), while organoid wall thickness significantly increased in organoids from pL2.Dclk1.N2fl mice (2d, measurements n = 47 pL2.Dclk1, n = 15 pL2.Dclk1.N2fl, n = 19 pL2.Dclk1.N2IC; 4d n = 39 pL2.Dclk1, n = 48 pL2.Dclk1.N2fl, n = 12 pL2.Dclk1.N2IC; 7d n = 30 pL2.Dclk1, n = 23 pL2.Dclk1.N2fl, n = 18 pL2.Dclk1.N2IC; ordinary two-way ANOVA, pL2.Dclk1 vs. pL2.Dclk1.N2fl p = 0.0011). (**C**) Representative brightfield pictures of pL2.Dclk1 cardia organoids undergoing the indicated treatments; scale bars = 100 µm. Organoid size was significantly reduced following treatment with DAPT (3d measurements n = 44 pL2.Dclk1, n = 38 pL2.Dclk1 DMSO, n = 19 pL2.Dclk1 DAPT, ordinary two-way ANOVA, pL2.Dclk1 vs. pL2.Dclk1 DAPT p = 0.041, pL2.Dclk1 DMSO vs. pL2.Dclk1 DAPT p = 0.015; 6d n = 98 pL2.Dclk1, n = 22 pL2.Dclk1 DMSO, n = 17 pL2.Dclk1 DAPT, ordinary two-way ANOVA, pL2.Dclk1 vs. pL2.Dclk1 DAPT p = 0.0005, pL2.Dclk1 DMSO vs. pL2.Dclk1 DAPT p = 0.0031); error bars indicate SEM.

Next, in order to investigate epithelial Notch signaling effects in the absence of a stromal niche, we employed cardia organoids from pL2.Dclk1, pL2.Dclk1.N2fl and pl2.Dclk1.N2IC mice. In line with our in vivo results, organoids from pL2.Dclk1.N2IC mice showed increased survival and growth compared to controls (Fig. 4A,B). While genetic Notch 2 receptor ablation did not change organoid survival and conferred only mildly decreased growth at 2 days compared to pL2.Dclk1 organoids (Fig. 4A,B), we observed increased thickening of the organoid wall, suggesting possible increased differentiation as observed in vivo (Fig. 4B). Previous studies have suggested that chronic inflammation mediated by IL-1b overexpression in the pL2 mouse model may act in paracrine fashion on epithelial cells to increase Notch signaling^3,10^. In line with paracrine activation of Notch signaling, DAPT treatment significantly reduced organoid growth in pL2.Dclk1 organoids (Fig. 4C)^31,32^. We recently verified the efficacy of DAPT treatment to reduce intracellular Notch signaling in a similar set of in vitro experiments^10^, and could similarly observe reduced Notch signaling in DAPT-treated pL2.Dclk1.N2IC organoids (Supplemental Fig. 4F). Taken together, the organoid data strongly suggest that, independent of stromal signaling, genetic or pharmacologic modulation of Notch signaling in gastric Dclk1-positive tuft cells is sufficient to alter epithelial turnover and differentiation.

### Notch activation in gastric Dclk1-positive tuft cells induces upregulation of oncogenic signaling

**Figure 5:**
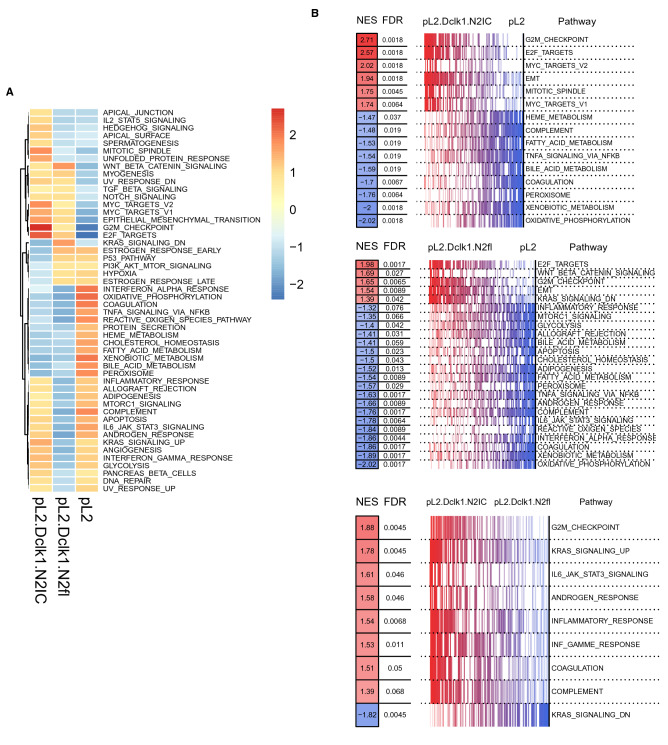
Notch activation in Dclk1-positive crypt epithelial cells induces upregulation of oncogenic signaling. (**A**) Analysis of HALLMARK gene sets (OneVsRest) of the indicated genotypes, scale bar indicates log2-fold changes of genes (Z-Scores; positive lgFC indicates higher gene expression in the respective group). (**B**) Grouped analyses of the indicated genotypes for changes in hallmark pathway gene sets, NES = normalized enrichment score, FDR = false discovery rate (red indicating increased, blue indicating decreased).

Finally, we aimed to study the transcriptomic landscapes underlying BE progression in pL2.Dclk1.N2IC mice and the increased secretory differentiation in pL2.Dclk1.N2fl SCJ tissues. To this end, we performed a microarray study of whole Barrett´s tissue taken from the SCJ of pL2.Dclk1.N2IC and pL2.Dclk1.N2fl mice induced at 6 months. Tissue was collected from study animals at 12 months, while tissues from age-matched L2-IL1B mice served as controls. Based on differentially expressed genes (DEG) between the groups, Gene Set Enrichment Analysis (GSEA) using Hallmark gene sets was performed and revealed a clear separation between the groups: the chronic inflammatory environment in pL2-IL1b mice induced a response to alpha interferon proteins, the upregulation of genes in response to reactive oxygen species, the prominent activation of IL-6/STAT3 and upregulated metabolism of bile acids and salts (Fig. 5A).

In contrast, pL2.Dclk1.N2IC tissue showed highly upregulated gene sets involved in the control of cellular division (E2F targets; G2M checkpoint; mitotic spindle) and oncogenic transformation (MYC targets; epithelial-mesenchymal-transition). We validated this by staining pL2.Dclk1.N2IC cardia tissues for c-Myc, which showed prominent expression compared to pL2.Dclk1 controls (Supplemental Fig. 5A). The oncogenic transcriptional landscape of pL2.Dclk1.N2IC tissues (Fig. 5B) is in line with the well-established pro-tumorigenic role of Notch signaling^33^. In addition, these transcriptomic changes corroborated our findings of significantly upregulated crypt fission events in tissues of pL2.Dclk1.N2IC mice. Lastly, gene expression analysis of pL2.Dclk1.N2fl tissues revealed upregulated Wnt signaling, along with upregulated genes known to be down-regulated by KRAS activation (Wnt beta catenin signaling; Kras signaling down). This was confirmed by analysis of β-Catenin nuclear translocation in cardia tissues of pL2.Dclk1.N2fl mice compared to pL2.Dclk1 controls (Supplemental Fig. 5B). The upregulation of Wnt signaling in pL2.Dclk1.N2fl tissues (Fig. 5B) corresponds well with its known role in directing secretory cell fate decisions^34,35^. Moreover, the prominent hallmark gene set “Kras_Signaling_Dn” would support the observed macroscopic phenotype of pL2.Dclk1.N2fl tissues, and suggests that genetic ablation of the Notch 2 receptor may lead to reduced oncogenic KRAS signaling.

## Discussion

Here, we have shown that Dclk1-positive tuft cells of the murine gastric cardia can become prominent modulators of BE development and progression, especially in response to alterations in Notch signaling. While Notch signaling is known to govern gastric and intestinal cell proliferation and differentiation^28,31^, Dclk1-positive gastric epithelial tuft cells are considered post-mitotic^18^. While a minority of intestinal tuft cells appears to express members of the Notch pathway^27^ indicating that these may be susceptible to modulations of Notch signaling, it appears surprising that the phenotype we observed in induced pL2.Dclk1.N2IC mice somewhat resembles the phenotype we previously observed in induced pL2.Lgr5.N2IC mice^10^, where intracellular Notch activation was targeted to well described gastric Lgr5-positive stem cells^36^. We hypothesize that the observed increase in both metaplasia and dysplasia in pL2.Dclk1.N2IC mice corresponds to the rather differentiated nature of gastric tuft cells, which may undergo dedifferentiation in this model, while Lgr5-positive gastric stem cells may undergo more distinct changes upon alterations in Notch signaling^10^. Lgr5-positive gastric cells expand during gastric tissue repair and metaplasia^3,36^, and a subset of small intestinal Lgr5-positive cells co-expressing *Dclk1* are described to behave as cancer stem cells^37^. Most gastric Dclk1-positive tuft cells co-stain for alpha gustducin or acetylated tubulin^22^, and we postulate that tuft cells of the cardia are similar to gastric tuft cells. However, we cannot entirely rule out that Lgr5-positive gastric stem cells acquiring *Dclk1* expression in the inflammatory environment of the pL2 model, or in the course of metaplastic changes following tamoxifen treatments^26^, contribute to the observed phenotypes.

The contribution of Dclk1-positive tuft cells to the accelerated onset of metaplasia and dysplasia in pL2.Dclk1.N2IC mice may originate from one of the following scenarios: First, the observed increase in crypt fission in cardia tissues from pL2.Dclk1.N2IC mice along with prominent tracing of metaplastic glands in pL2.Dclk1.N2IC.LacZ mice, might suggest the conversion of Dclk1-positive epithelial cells into a progenitor phenotype. Activation of Notch signaling has been shown to induce crypt fission in the physiological context of tissue development^28^, and Notch activation in gastric Lgr5-positive stem cells is sufficient to induce a marked increase in gastric fission events and promote clonal stem cell expansion^38^. Notch activation results in downstream propagation of Myc signaling^39^, which appears to promote crypt fission^40^, and we readily detected increased HALLMARK_MYC_TARGET signaling in pL2.Dclk1.N2IC tissues. In this regard, crypt fissions appear as a mechanism rather specific to cells with stem cell features to enable propagation of stem cells, without necessarily affecting tissue proliferation^41^. This could correspond to the model of reserve stem cells and the concept of plasticity, with reprogrammed post-mitotic cells contributing to tissue regeneration^42^. In fact, the reversal of a differentiated cell type into a more progenitor-like phenotype following injury has recently been proposed as a fundamental process termed paligenosis to promote tissue regeneration^43^, Interestingly, in this model acute injury mediated by high doses of tamoxifen induced the rapid decrease of mTORC1 in gastric differentiated cells which enabled their dedifferentiation, with subsequent upregulation of mTORC1 required for injury-induced proliferation^43^. In this regard, the experimental setup employed in our studies could induce a similar mechanism, and activated Notch signaling synergize with upregulated mTORC1 in dedifferentiated tuft cells^44^. In addition, the prominent decrease of DCLK1-positive tuft cells in pL2.Dclk1.N2fl tissues correlates with the decelerated phenotype, supporting the contribution of tuft cells to BE formation and further pointing at the putative importance of N2 for their regeneration following injury^45^.

If this reprogramming occurred in long-lived cells, however, it may unmask oncogenic mutations leading to tumor growth^42^. Interestingly, intestinal Dclk1-positive tuft cells have been proposed to be long-lived, and indeed inflammation in conjunction with an oncogenic mutation specific to Dclk1-positive tuft cells caused tumor formation in a mouse model of colorectal cancer^22^. In light of the substantial mutational load described in BE tissues^46^, the chronic inflammatory environment of the pL2 mouse model combined with increased Notch signaling may thus suffice to induce oncogenic growth from Dclk1-positive tuft cells. Lastly, *Dclk1* is also expressed in cells of the enteric nervous system (ENS)^21,47^, and modulations of Notch signaling in cells of the ENS may result in an altered glial and neuronal differentiation^48,49^ and contribute to the epithelial phenotype.

On the other hand, Dclk1-positive tuft cells stand out in comparison to other differentiated cell types, in that these appear to represent important contributors to the intestinal niche^19^. The functional importance of their abundance within the gastric cardia^50^, and their contribution to BE development in particular, remain poorly understood. However, tuft cells are chemosensory cells, and in the context of the cardia it stands out that these cells appear well equipped to sense changes to gastric and bile acid^19^. Indeed, tuft cells appear similarly abundant in the rat common bile duct as in the cardia^51^. Bile acids have been identified to accelerate the development of Barret-like metaplasia, and given the prominent expansion of Dclk1-positive tuft cells in the cardia of murine and human Barrett´s metaplasia^3^ in conjunction with the data presented in this study it appears feasible to postulate an important modulatory role of tuft cells in BE formation. Furthermore, given that tuft cells appear morphologically well equipped to exert epithelial paracrine signaling^52^, their unique expression of enzymes such as COX-1 and COX-2 may represent an additional means to promote BE development^18,53^. Indeed, BE and EAC tissues showed elevated levels of COX2, and pharmaceutical blockade of the COX2-TXA2 pathway in some studies led to reduced development of BE and EAC^54^. Furthermore, while our data did not reveal changes to the studied immune cell populations, intestinal tuft cells appear to closely communicate with innate lymphoid cells 2 (ILC2)^13–15^ and innate lymphoid cell signaling may play a role in the inflammatory environment of BE^55^. In addition to other stromal cell types tuft cells communicate with^19^, Notch-mediated activation of gastric Dclk1-positive tuft cells may therefore result in the initiation of pro-tumorigenic niche signaling.

Dclk1-positive epithelial tuft cells have been observed in the columnar but not squamous mucosa throughout the gastrointestinal tract^19,22^. The cellular origin of Barrett´s esophagus remains a matter of debate, as squamous^7,8^ as well as columnar^3,10^ cell types have been suggested to give rise to Barrett-like lesions. While our data here shows the potential contribution of columnar Dclk1-positive cells to BE development and progression, the model also supports the notion that post-mitotic cells can contribute to oncogenic tissue transformation, in part by undergoing cellular reprogramming^42^. The fact that cardia tissues from pL2.Dclk1.N2fl mice, with genetic deletion of Notch receptor signaling, showed a markedly reduced BE phenotype, supports the notion that progression to EAC is likely dependent on the activation of multiple oncogenic pathways in combination with an inflammatory environment. Indeed, our microarray data revealed possible synergistic oncogenic signaling of activated Notch and KRAS, as the direct comparison of pL2.Dclk1.N2IC vs pL2.Dclk1.N2fl tissues showed strong upregulation of the hallmark gene set “Kras_Signaling_Up”. Similar prominent synergy of Notch and KRAS signaling has been shown to promote pancreatic neoplastic tissue transformation^56^. In conclusion, together with the accelerated BE progression in pL2.Lgr5.N2IC mice^10^, our data strongly support the importance of Notch signaling for BE development and progression.

## Materials and methods

### Mouse models

The genetic mouse model (pL2-IL1b) that overexpresses Interleukin-1b in the mouse esophagus and stomach^3^ as well as the Dclk1-CreERT2^22^, Lgr5-EGFP-IRES-CreERT2^57^ Notch2-IC^24^, Notch2 flox/flox^25^ and Rosa26-LacZ^58^ mouse models have been described recently. Mice were inter-crossed to obtain the genotypes pL2-IL1b;Dclk1-CreERT2 (pL2.Dclk1), pL2-IL1b;Dclk1-CreERT2;R26-LacZ (pL2.Dclk1.LacZ), pL2-IL1b;Dclk1-CreERT2;N2IC^F/F^ (pL2.Dclk1.N2IC), pL2-IL1b;Dclk1-CreERT2;N2IC^F/F^;R26-LacZ (pL2.Dclk1.N2IC.LacZ) and pL2-IL1b;Dclk1-CreERT2;N2^F/F^ (pL2.Dclk1.N2fl) mice. Tamoxifen (6 mg, T5648-5G, Sigma) was administered to mice of the respective genotype at 6 months of age in three separate administrations within one week. Efficient recombination of the Dclk1-CreERT2 transgene was verified previously^22^, and the employed tamoxifen regimen ensured comparability to our previous studies^10,22^. As the high dose of tamoxifen may result in parietal cell loss and metaplasia of chief cells^26,59^, all comparison groups received the same tamoxifen treatment. Genotyping was routinely performed. All animal experiments were approved by the ethics committee of the District Government of Upper Bavaria (TVA 55.2-1-54-2532-44-12) and performed in compliance with the ARRIVE guidelines and the German Animal Welfare and Ethical Guidelines of the Klinikum rechts der Isar, TUM, Munich, Germany.

### Tissue preparation and disease evaluation

For macroscopic scoring, the stomach was opened along the large curvature and flattened for documentation. Macroscopic scoring of the squamocolumnar junction (SCJ) and the esophagus was performed and scores averaged as shown previously^10^. For histopathologic evaluation, mouse tissues were fixed in formalin and paraffin-embedded, cut and stained with H&E (haematoxylin and eosin). Histopathological scores were performed by an experienced mouse pathologist by previously established criteria for the influx of immune cells per high-power field, metaplasia and dysplasia^60^. Inflammation was scored by the percentage of different immune cells in a defined tissue area of the SCJ in a high-power field evaluation. Metaplasia was evaluated by the abundance of mucus producing or cells per gland and the abundance of glands with mucus producing cells in the BE area at the SCJ. Dysplasia was evaluated by the amount of cellular atypia and the presence of low or high grade dysplasia in single or multiple glands as assessed by experienced mouse pathologists. Mucus production was assessed by Periodic Acid-Schiff-(PAS) staining and quantified as percentage of PAS positive area in BE regions. Crypt fission was quantified by counting fused crypts in the BE region similar to a previously described method^61^.

### Immunohistochemistry and immunofluorescence

Standard immunohistochemical procedures with citrate buffer antigen retrieval (1.00244.1000, Merck) were performed using following antibodies: primary rat anti-human Notch2-IC antibody^62^ (DSHB Hybridoma Bank, C651.6DbHN-c, 1:500; overnight at 4 °C), rabbit anti-mouse DCLK1 antibody (Abgent, AP7219b), rabbit anti-mouse c-Myc (Abcam, ab32072), mouse anti-mouse β-Catenin (BD Biosciences, 610154), and secondary anti-rat (Vector Labs, BA-4000), anti-rabbit (Vector Labs, BA-1000) and anti-mouse (Vector Labs, BA-9200) antibody. For immunofluorescence, stainings were performed as recently described^17^. The following antibodies were used: rat anti-Ki67 (Invitrogen, 14-5698-82), secondary goat anti-rat Alexa 488 (Invitrogen, A11006) and goat anti-rabbit Alexa 594 (Invitrogen, A11037). Quantification was assessed as percentage of positive cells or areas within BE regions as previously described^60^. Determination of beta-gal activity was achieved as described previously^22^.

### Organoid culture

The cardia and forestomach tissue of mice was extracted for organoid culture as previously described^47^. The respective transgenic mice were induced in vivo as outlined above and tissue for culture collected following induction. At least three independent primary organoid lines where freshly isolated from each indicated transgenic mouse line, organoids were used for cell proliferation or differentiation experiments only in early passages during optimal expansion rates as previously described^10^. Cells were exposed to small molecule inhibitor DAPT (D5942, Sigma, 50 µM) for 72 h to block Notch signaling. Organoid survival was microscopically assessed according to the number of organoids two days after isolation relative to those with a viable morphology at day seven after isolation. Organoid growth and size was evaluated according to microscopic analyses.

### Real-time PCR analysis

Quantitative PCR was performed using the LightCycler 480 Instrument (Roche) and the QuantiFast SYBR Green PCR Kit (4000) (204057, Qiagen) according to the manufacturer’s instructions. RNA levels were normalized to *GAPDH* levels. The primer sequences for *Notch2* are as follows: Fwd CCCAGAACCAATCAGGTTAGC, Rv GCCGAGACTCTAGCAATCACAA.

### Microarray study design and analysis

Tissues from the SCJ of mice with the genotype pL2-IL1b (n = 3), pL2.Dclk1.N2IC (n = 3) and pL2.Dclk1.N2fl (n = 3) 6 months following induction (induced at 6 months; aged 12 months at sacrifice) were subjected to RNA extraction and amplification using the Ambion WT expression kit (Thermo Fisher). The whole-transcriptome array GeneChip Mouse Gene ST Array (Affymetrix) 2.1st was employed for transcriptome analysis. RTA (Illumina) was used for base calling and bcl2fastq2 (version 2.20) for converting BCL to fastq format, coupled with adaptor trimming. Pseudoalignment was carried out to a kallisto index created from the murine GRCm38 transcriptome using kallisto (0.44.0). Estimated counts and transcripts per kilobase million (TPM) per gene were computed from the kallisto output using the tximport R package^63^. To test for DEG between L2-IL1B and Dclk1-transgenic mice a negative binomial generalized linear model was used as implemented in the DESeq2 R package^64^ and a false discovery rate < 0.1 was considered significant. The expression in TPM for select genes was illustrated in a heatmap using the pheatmap R package (https://CRAN.R-project.org/package=pheatmap).

### Statistical analysis

Analyses were performed using GraphPad Prism Software. Statistical measures include mean values, standard error of the mean, multiple t-test calculations corrected for nonparametric testing or ordinary two-way ANOVA analyses. Outliers were excluded from statistical analysis. Statistical details of an experiment can be found in the figure legends. Statistical significance is defined for p values < 0.05 between groups.

## Supplementary Information

Supplementary Legends.

Supplementary Figures.

## Data Availability

The authors declare that all data supporting the findings of this study are available within the article or from the corresponding author upon reasonable request. The RNA-sequencing data reported in this study are available from Gene Expression Omnibus with accession code GSE158116.

## References

[1] Spechler SJ, Souza RF (2014). Barrett's esophagus. N. Engl. J. Med..

[2] Nakagawa H (1997). The targeting of the cyclin D1 oncogene by an Epstein-Barr virus promoter in transgenic mice causes dysplasia in the tongue, esophagus and forestomach. Oncogene.

[3] Quante M (2012). Bile acid and inflammation activate gastric cardia stem cells in a mouse model of Barrett-like metaplasia. Cancer Cell.

[4] Van Es JH (2005). Notch/gamma-secretase inhibition turns proliferative cells in intestinal crypts and adenomas into goblet cells. Nature.

[5] Kim TH, Shivdasani RA (2011). Notch signaling in stomach epithelial stem cell homeostasis. J. Exp. Med..

[6] Reedijk M (2008). Activation of Notch signaling in human colon adenocarcinoma. Int. J. Oncol..

[7] Nicholson AM (2012). Barrett's metaplasia glands are clonal, contain multiple stem cells and share a common squamous progenitor. Gut.

[8] Jiang M (2017). Transitional basal cells at the squamous-columnar junction generate Barrett's oesophagus. Nature.

[9] Leedham SJ (2008). Individual crypt genetic heterogeneity and the origin of metaplastic glandular epithelium in human Barrett's oesophagus. Gut.

[10] Kunze B (2020). Notch Signaling Mediates Differentiation in Barrett's Esophagus and Promotes Progression to Adenocarcinoma. Gastroenterology.

[11] Giannakis M (2006). Molecular properties of adult mouse gastric and intestinal epithelial progenitors in their niches. J. Biol. Chem..

[12] Gagliardi G, Moroz K, Bellows CF (2012). Immunolocalization of DCAMKL-1, a putative intestinal stem cell marker, in normal colonic tissue. Pathol. Res. Pract..

[13] Von Moltke J, Ji M, Liang HE, Locksley RM (2016). Tuft-cell-derived IL-25 regulates an intestinal ILC2-epithelial response circuit. Nature.

[14] Gerbe F (2016). Intestinal epithelial tuft cells initiate type 2 mucosal immunity to helminth parasites. Nature.

[15] Howitt MR (2016). Tuft cells, taste-chemosensory cells, orchestrate parasite type 2 immunity in the gut. Science.

[16] Wilen CB (2018). Tropism for tuft cells determines immune promotion of norovirus pathogenesis. Science.

[17] Middelhoff M (2020). Prox1-positive cells monitor and sustain the murine intestinal epithelial cholinergic niche. Nat. Commun..

[18] Gerbe F (2011). Distinct ATOH1 and Neurog3 requirements define tuft cells as a new secretory cell type in the intestinal epithelium. J. Cell Biol..

[19] Middelhoff M (2017). Dclk1-expressing tuft cells: Critical modulators of the intestinal niche?. Gastrointestinal Liver Physiol..

[20] Bailey JM (2014). DCLK1 marks a morphologically distinct subpopulation of cells with stem cell properties in preinvasive pancreatic cancer. Gastroenterology.

[21] Hayakawa Y (2017). Nerve growth factor promotes gastric tumorigenesis through aberrant cholinergic signaling. Cancer Cell.

[22] Westphalen CB (2014). Long-lived intestinal tuft cells serve as colon cancer-initiating cells. J. Clin. Investig..

[23] Chandrakesan P (2014). DCLK1 facilitates intestinal tumor growth via enhancing pluripotency and epithelial mesenchymal transition. Oncotarget.

[24] Hampel F (2011). CD19-independent instruction of murine marginal zone B-cell development by constitutive Notch2 signaling. Blood.

[25] Besseyrias V (2007). Hierarchy of Notch-Delta interactions promoting T cell lineage commitment and maturation. J. Exp. Med..

[26] Huh WJ (2012). Tamoxifen induces rapid, reversible atrophy, and metaplasia in mouse stomach. Gastroenterology.

[27] Haber AL (2017). A single-cell survey of the small intestinal epithelium. Nature.

[28] Demitrack ES, Samuelson LC (2017). Notch as a driver of gastric epithelial cell proliferation. Cell Mol. Gastroenterol. Hepatol..

[29] Fitzgerald RC (2002). Inflammatory gradient in Barrett's oesophagus: Implications for disease complications. Gut.

[30] Miele L (2006). Notch signaling. Clin. Cancer Res..

[31] VanDussen KL (2012). Notch signaling modulates proliferation and differentiation of intestinal crypt base columnar stem cells. Development.

[32] Menke V (2010). Conversion of metaplastic Barrett's epithelium into post-mitotic goblet cells by gamma-secretase inhibition. Dis. Model Mech..

[33] Yuan X (2015). Notch signaling: An emerging therapeutic target for cancer treatment. Cancer Lett..

[34] Van Es JH (2005). Wnt signalling induces maturation of Paneth cells in intestinal crypts. Nat. Cell Biol..

[35] Andreu P (2005). Crypt-restricted proliferation and commitment to the Paneth cell lineage following Apc loss in the mouse intestine. Development.

[36] Leushacke M (2017). Lgr5-expressing chief cells drive epithelial regeneration and cancer in the oxyntic stomach. Nat. Cell Biol..

[37] Nakanishi Y (2013). Dclk1 distinguishes between tumor and normal stem cells in the intestine. Nat. Genet..

[38] Demitrack ES (2015). Notch signaling regulates gastric antral LGR5 stem cell function. EMBO J..

[39] Huang T (2016). NOTCH receptors in gastric and other gastrointestinal cancers: Oncogenes or tumor suppressors?. Mol. Cancer.

[40] Muncan V (2006). Rapid loss of intestinal crypts upon conditional deletion of the Wnt/Tcf-4 target gene c-Myc. Mol. Cell. Biol..

[41] Hirata A (2013). Dose-dependent roles for canonical Wnt signalling in de novo crypt formation and cell cycle properties of the colonic epithelium. Development.

[42] Mills JC, Sansom OJ (2015). Reserve stem cells: Differentiated cells reprogram to fuel repair, metaplasia, and neoplasia in the adult gastrointestinal tract. Sci. Signal.

[43] Willet SG (2018). Regenerative proliferation of differentiated cells by mTORC1-dependent paligenosis. EMBO J..

[44] Ma J (2010). Mammalian target of rapamycin regulates murine and human cell differentiation through STAT3/p63/Jagged/Notch cascade. J. Clin. Investig..

[45] Carulli AJ (2015). Notch receptor regulation of intestinal stem cell homeostasis and crypt regeneration. Dev. Biol..

[46] Ross-Innes CS (2015). Whole-genome sequencing provides new insights into the clonal architecture of Barrett's esophagus and esophageal adenocarcinoma. Nat. Genet..

[47] Pastula A (2016). Three-dimensional gastrointestinal organoid culture in combination with nerves or fibroblasts: A method to characterize the gastrointestinal stem cell niche. Stem Cells Int..

[48] McCallum S (2020). Enteric glia as a source of neural progenitors in adult zebrafish. Elife.

[49] Morrison SJ (2000). Transient Notch activation initiates an irreversible switch from neurogenesis to gliogenesis by neural crest stem cells. Cell.

[50] Hass N, Schwarzenbacher K, Breer H (2007). A cluster of gustducin-expressing cells in the mouse stomach associated with two distinct populations of enteroendocrine cells. Histochem. Cell Biol..

[51] Sato A (2007). Tuft cells. Anat. Sci. Int..

[52] Hoover B (2017). The intestinal tuft cell nanostructure in 3D. Sci. Rep..

[53] Bezencon C (2008). Murine intestinal cells expressing Trpm5 are mostly brush cells and express markers of neuronal and inflammatory cells. J. Comp. Neurol..

[54] Zhang T (2019). Targeting the COX1/2-Driven thromboxane A2 pathway suppresses Barrett's esophagus and esophageal adenocarcinoma development. EBioMedicine.

[55] Karstens KF (2020). Anti-inflammatory microenvironment of esophageal adenocarcinomas negatively impacts survival. Cancer Immunol.. Immunother..

[56] De La OJ (2008). Notch and Kras reprogram pancreatic acinar cells to ductal intraepithelial neoplasia. Proc. Natl. Acad. Sci. USA.

[57] Barker N (2007). Identification of stem cells in small intestine and colon by marker gene Lgr5. Nature.

[58] Soriano P (1999). Generalized lacZ expression with the ROSA26 Cre reporter strain. Nat. Genet..

[59] Stange DE (2013). Differentiated Troy+ chief cells act as reserve stem cells to generate all lineages of the stomach epithelium. Cell.

[60] Fox JG (2000). Concurrent enteric helminth infection modulates inflammation and gastric immune responses and reduces helicobacter-induced gastric atrophy. Nat. Med..

[61] Jin G (2009). Inactivating cholecystokinin-2 receptor inhibits progastrin-dependent colonic crypt fission, proliferation, and colorectal cancer in mice. J. Clin. Investig..

[62] Zagouras P, Stifani S, Blaumueller CM, Carcangiu ML, Artavanis-Tsakonas S (1995). Alterations in Notch signaling in neoplastic lesions of the human cervix. Proc. Natl. Acad. Sci. USA.

[63] Soneson C, Love MI, Robinson MD (2015). Differential analyses for RNA-seq: Transcript-level estimates improve gene-level inferences. F1000Res.

[64] Love MI, Huber W, Anders S (2014). Moderated estimation of fold change and dispersion for RNA-seq data with DESeq2. Genome Biol.

