# Supplementary material for: Notch signaling drives development of Barrett’s metaplasia from Dclk1-positive epithelial tuft cells in the murine gastric mucosa
Source: Sci Rep. 2021 Feb 24;11:4509. doi: 10.1038/s41598-021-84011-4 (PMC7904766; doi:10.1038/s41598-021-84011-4)
Supplement: Supplementary file 1 — Supplementary Legends. [file 41598_2021_84011_MOESM1_ESM.docx]

**Supplemental Figures**

**Fig. S1. The pL2 mouse model mimics human BE pathology.** (A) Experimental scheme illustrating timepoints of tamoxifen induction and tissue collection for analysis. (B) Representative H&E pictures of the SCJ of WT and pL2.Dclk1 mice, respectively; for pL2.Dclk1, at 6+3 and 6+6 the same pictures are shown as in Fig. 1B for comparison to WT; scale bars=200µm. (C) Histopathologic scoring in pL2.Dclk1 compared to WT controls for metaplasia (6+3 n=5 WT, n=6 pL2.Dclk1 mice, ordinary two-way ANOVA, p=<0.0001; 6+6 n=6 WT, n=5 pL2.Dclk1 mice, ordinary two-way ANOVA, p=<0.0001; 6+9 n=4 WT, n=8 pL2.Dclk1 mice, ordinary two-way ANOVA, p=0.0001) and dysplasia (6+3 n=5 WT, n=6 pL2.Dclk1 mice, ordinary two-way ANOVA, p=0.038; 6+6 n=6 WT, n=5 pL2.Dclk1 mice; 6+9 n=4 WT, n=8 pL2.Dclk1 mice). (D) Histopathologic scoring in pL2.Dclk1 tissues compared to WT controls for acute inflammation (6+3 n=5 WT, n=6 pL2.Dclk1 mice, ordinary two-way ANOVA, p=0.0005; 6+6 n=6 WT, n=6 pL2.Dclk1 mice, ordinary two-way ANOVA, p=0.001; 6+9 n=4 WT, n=8 pL2.Dclk1 mice, ordinary two-way ANOVA, p=0.002). (E) Survival analysis of pL2.Dclk1 mice compared to WT controls (n=25 WT; n=31 pL2.Dclk1; Median survival WT and pL2.Dclk1=>10 months; log-rank (Mantel-Cox) test, Chi square=8.745, p=0.0031). (F) Weight analyses of pL2.Dclk1 mice compared to WT controls showed substantial decreases in overall weight in both male and female mice (male total n=6 WT; total n=26 pL2.Dclk1; female total n=17 WT; total n=17 pL2.Dclk1); error bars indicate SEM.

**Fig. S2. Tamoxifen administration induces expansion of DCLK1-positive tuft cells.** (A) Representative stainings for DCLK1 of the SCJ of pL2-IL1b and pL2.Dclk1 mice, respectively; scale bars=200µm; insets scale bar=50µm. (B) Quantification of DCLK1 staining in pL2.Dclk1 tissues compared to pL2-IL1b controls (n=3 pL2-IL1b 9mo, n=3 pL2.Dclk1 6+3 mice, unpaired t test, p=0.047). (C) Representative stainings for Notch2-IC stained nuclei of the SCJ of pL2-IL1b and pL2.Dclk1 mice, respectively; scale bars=200µm; insets scale bar=50µm. (D) Quantification of Notch2-IC staining in pL2.Dclk1 tissues compared to pL2-IL1b controls (n=3 pL2-IL1b 9mo, n=3 pL2.Dclk1 6+3 mice); error bars indicate SEM.

**Fig. S3. Notch activation in Dclk1-positive gastric tuft cells accelerates BE development.** (A) Analysis of Notch2 mRNA expression in pL2.Dclk1.N2IC tissues compared to pL2.Dclk1 controls (n=8 pL2.Dclk1.N2IC 6+6; n=7 pL2.Dclk1 6+6 controls; Wilcoxon matched-pairs signed rank test, p=0.016). (B) Representative staining for LacZ of the SCJ of Dclk1-CreERT2; R26-LacZ control mice; scale bar=100µm. (C) Representative stainings for LacZ of the SCJ of induced pL2.Dclk1.LacZ and pL2.Dclk1.N2IC.LacZ mice, respectively; dashed line indicates SCJ, scale bar=200µm. (D) Representative co-stainings for DCLK1 and Ki67 of the SCJ of induced pL2.Dclk1 (6+3) and pL2.Dclk1.N2IC (6+6) mice, respectively; white arrowheads show positive overlap near the SCJ (delineated by dashed line); scale bars=100µm. (E) Representative staining for DCLK1 in a Lgr5-EGFP-IRES-CreERT2 reporter mouse shows no positive overlap near the SCJ (delineated by dashed line); scale bar=100µm. (F) Weight analyses of pL2.Dclk1.N2IC compared to pL2.Dclk1 controls showed substantial decreases in overall weight in both male and female mice (male total n=31 pL2.Dclk1.N2IC; total n=26 pL2.Dclk1 controls; female total n=38 pL2.Dclk1.N2IC; total n=17 pL2.Dclk1 controls). (G) Histopathologic scoring in pL2.Dclk1.N2IC mice compared to pL2.Dclk1 controls for acute inflammation (6+3 n=7 pL2.Dclk1, n=25 pL2.Dclk1.N2IC mice; 6+6 n=6 pL2.Dclk1, n=8 pL2.Dclk1.N2IC mice); error bars indicate SEM.

**Fig. S4. Genetic ablation of the Notch 2 receptor in Dclk1-positive gastric cells decelerates BE development.** (A) Representative macroscopic pictures of the opened stomach of pL2.Dclk1 and pL2.Dclk1.N2fl mice, respectively; scale bars=0.5cm. (B) Macroscopic scoring in pL2.Dclk1.N2fl mice compared to pL2.Dclk1 controls (6+3 n=36 pL2.Dclk1.N2fl, n=14 pL2.Dclk1 controls; 6+6 n=32 pL2.Dclk1.N2fl, n=8 pL2.Dclk1 controls; 6+9 n=8 pL2.Dclk1.N2fl, n=12 pL2.Dclk1 controls, ordinary two-way ANOVA, p=0.024). (C) Representative stainings for DCLK1 of the SCJ of pL2.Dclk1 and pL2.Dclk1.N2fl mice, respectively, and quantification of DCLK1 staining (n=3 pL2.Dclk1, n=3 pL2.Dclk1.N2fl mice); for pL2.Dclk1, the same image and quantification data is shown as in Fig. S2A and B for comparison to pL2.Dclk1.N2fl tissue; scale bars=200µm; insets scale bar=50µm. (D) Histopathologic scoring in pL2.Dclk1.N2fl mice compared to pL2.Dclk1 controls for metaplasia (6+3 n=6 pL2.Dclk1.N2fl, n=7 pL2.Dclk1 controls; 6+6 n=9 pL2.Dclk1.N2fl, n=5 pL2.Dclk1 controls; 6+9 n=6 pL2.Dclk1.N2fl, n=8 pL2.Dclk1 controls) and dysplasia (6+3 n=6 pL2.Dclk1.N2fl, n=7 pL2.Dclk1 controls; 6+6 n=8 pL2.Dclk1.N2fl, n=5 pL2.Dclk1 controls; 6+9 n=6 pL2.Dclk1.N2fl, n=8 pL2.Dclk1 controls). (E) Survival analysis of pL2.Dclk1 controls compared to pL2.Dclk1.N2fl mice (n=31 pL2.Dclk1; n=41 pL2.Dclk1.N2fl; Median survival pL2.Dclk1 and pL2.Dclk1.N2fl=>10 months). (F) Representative pictures of cardia organoids of pL2.Dclk1.N2IC mice stained for Notch2-IC; pL2.Dclk1.N2IC DMSO scale bar=50µm, pL2.Dclk1.N2IC DAPT scale bar=100µm; error bars indicate SEM.

**Fig. S5. Notch signaling in Dclk1-positive tuft cells modulates intracellular c-Myc and Beta Catenin signaling.** (A) Representative stainings for c-Myc of the SCJ of pL2-IL1b and pL2.Dclk1.N2IC mice, respectively; scale bar=100µm. (B) Representative stainings for Beta Catenin of the SCJ of pL2-IL1b and pL2.Dclk1.N2fl mice, respectively; scale bar=100µm; dashed line delineates SCJ.
