# Supplementary material for: Notch signaling drives development of Barrett’s metaplasia from Dclk1-positive epithelial tuft cells in the murine gastric mucosa
Source: Sci Rep. 2021 Feb 24;11:4509. doi: 10.1038/s41598-021-84011-4 (PMC7904766; doi:10.1038/s41598-021-84011-4)

Notch signaling drives development of Barrett´s metaplasia from Dclk1-positive epithelial tuft cells in the murine gastric mucosa

Bettina Kunze[1, *], Moritz Middelhoff[1, *, #], H. Carlo Maurer[1], Tatiana Agibalova[1], Akanksha Anand[1], Anne-Marie Bührer[1], Hsin-Yu Fang[1], Theresa Baumeister[1], Katja Steiger[2], Julia Strangmann[1], Roland M. Schmid[1], Timothy C. Wang[3], Michael Quante[1, 4, #]

[1] Klinik und Poliklinik für Innere Medizin II, Technical University of Munich, Munich, Germany

[2] Institute of Pathology, Technical University of Munich, Munich, Germany

[3] Department of Medicine, Columbia University Medical Center, New York, NY, USA

[4] Klinik für Innere Medizin II, Gastrointestinale Onkologie, Universitätsklinikum Freiburg, Freiburg, Germany

* these authors contributed equally

[#] Corresponding authors:

Moritz Middelhoff, MD

Mail: moritz.middelhoff@tum.de

Michael Quante, MD

Mail: michael.quante@uniklinik-freiburg.de

Supplemental Figure 1

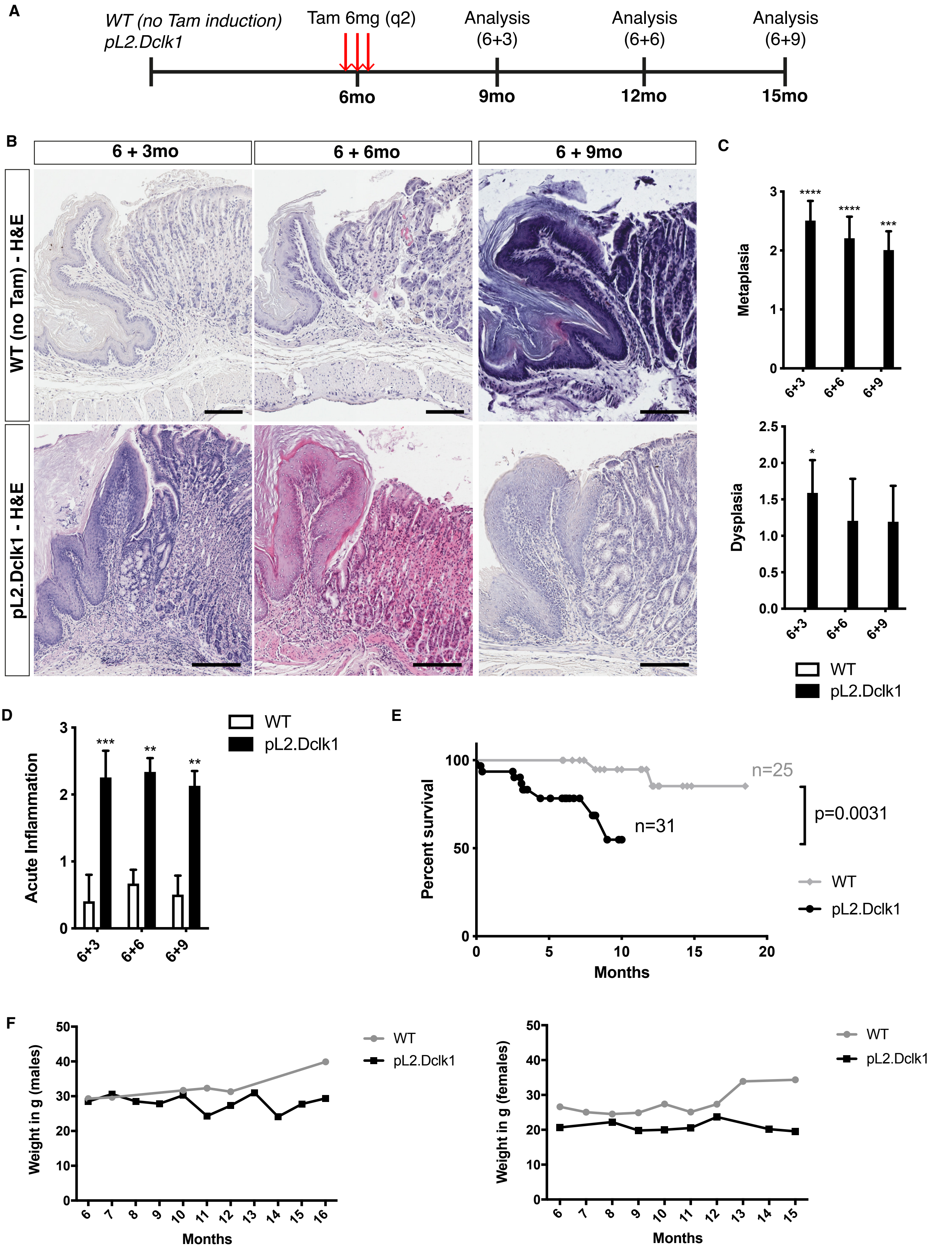

Supplemental Figure 2

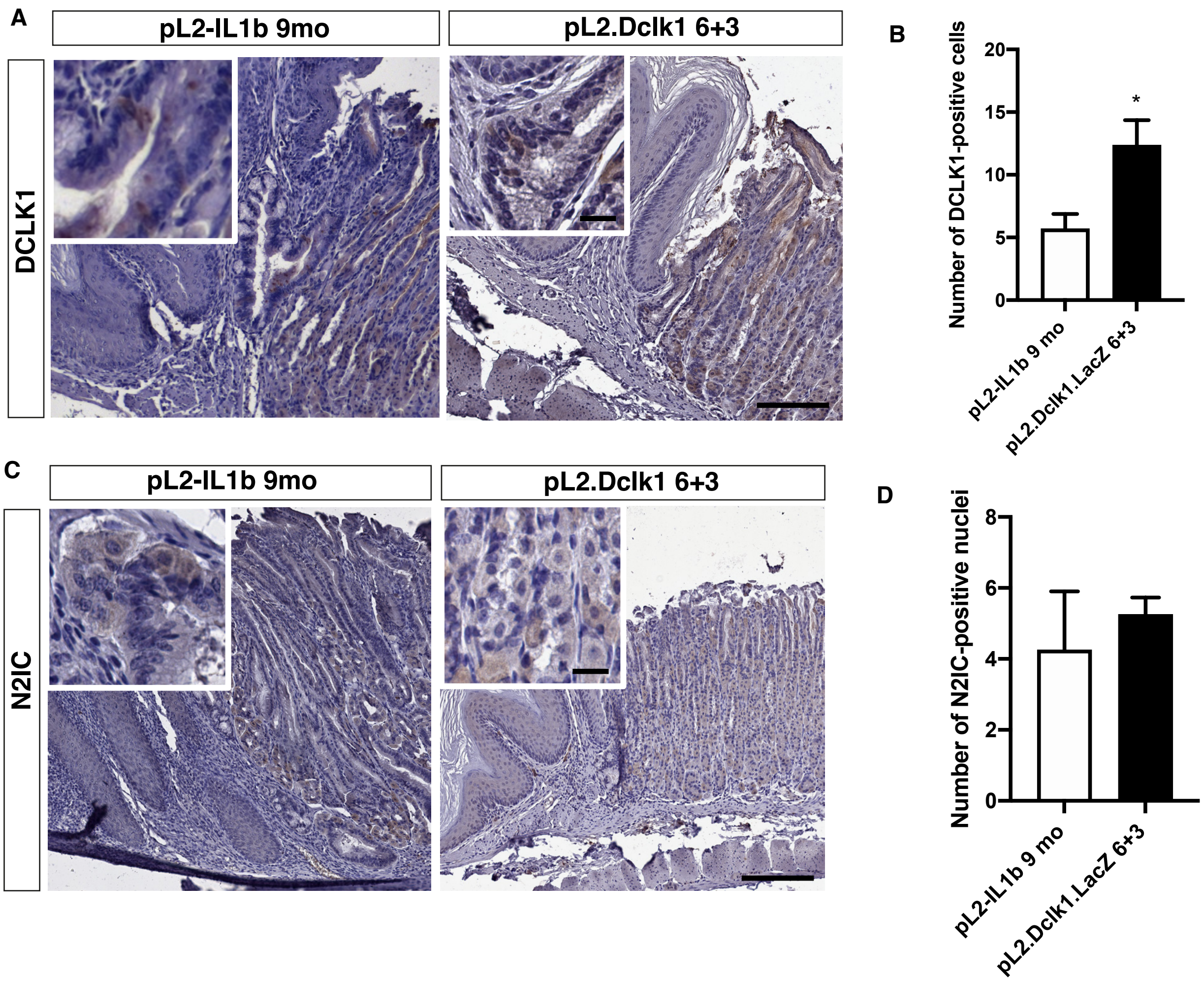

Supplemental Figure 3

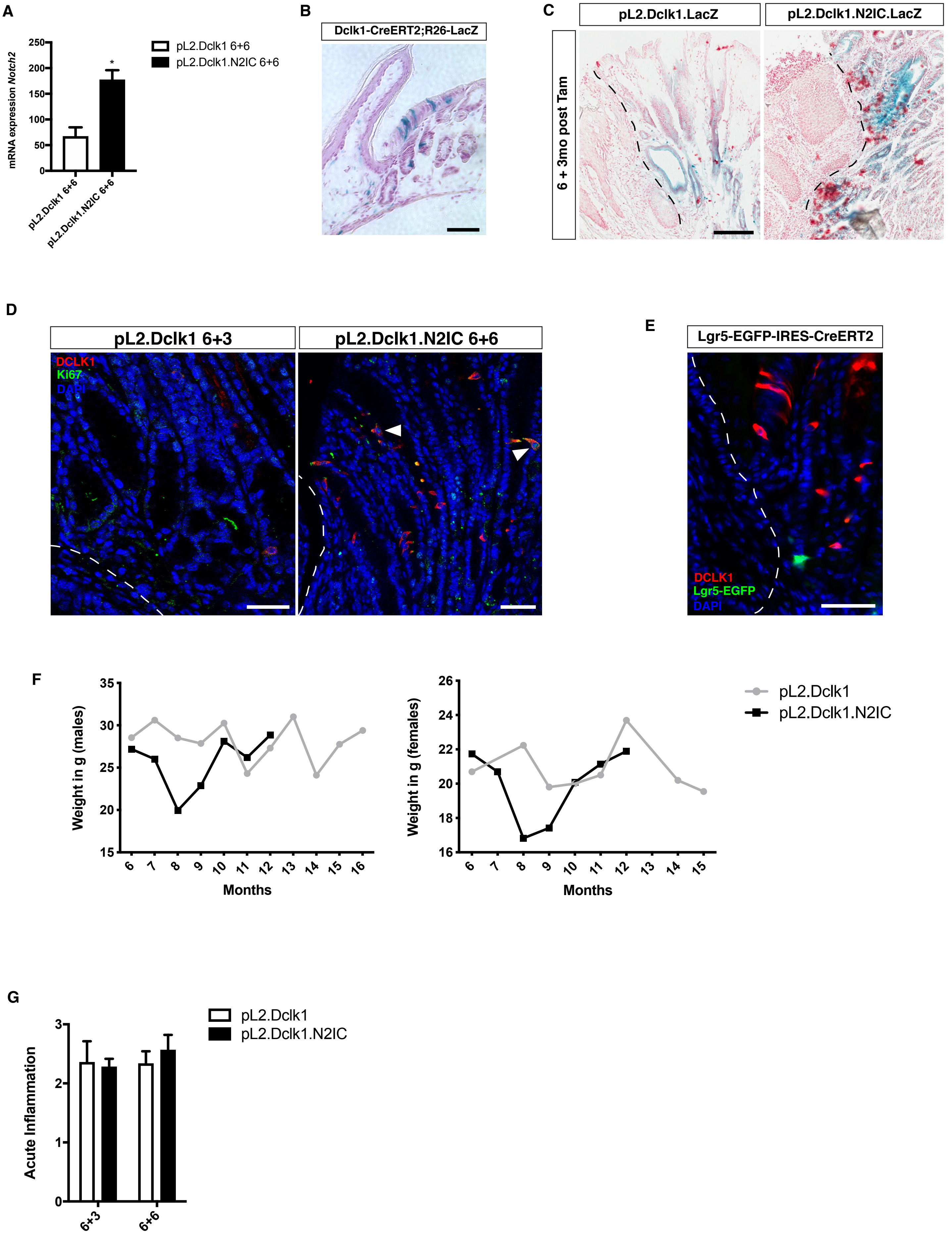

Supplemental Figure 4

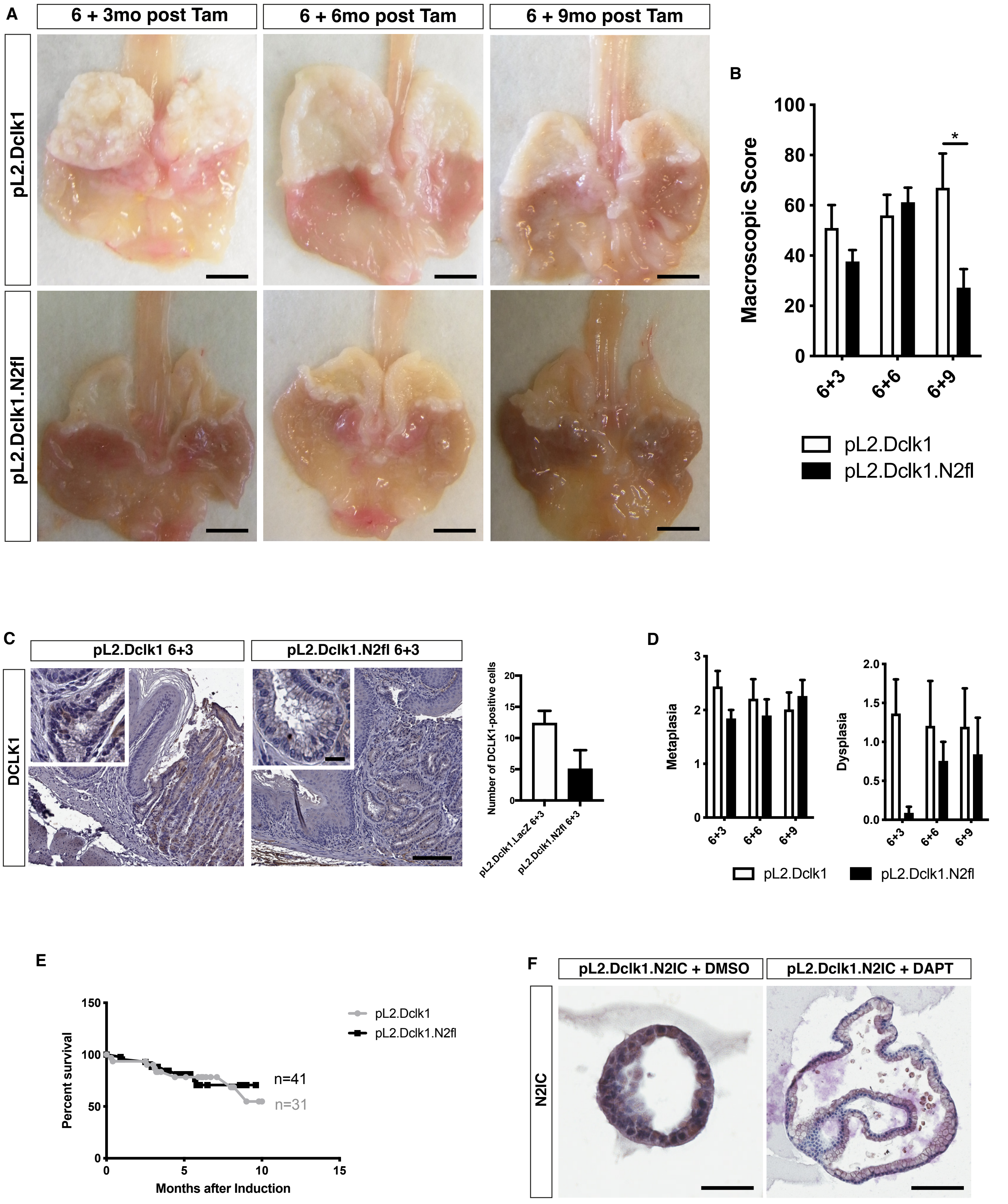

Supplemental Figure 5

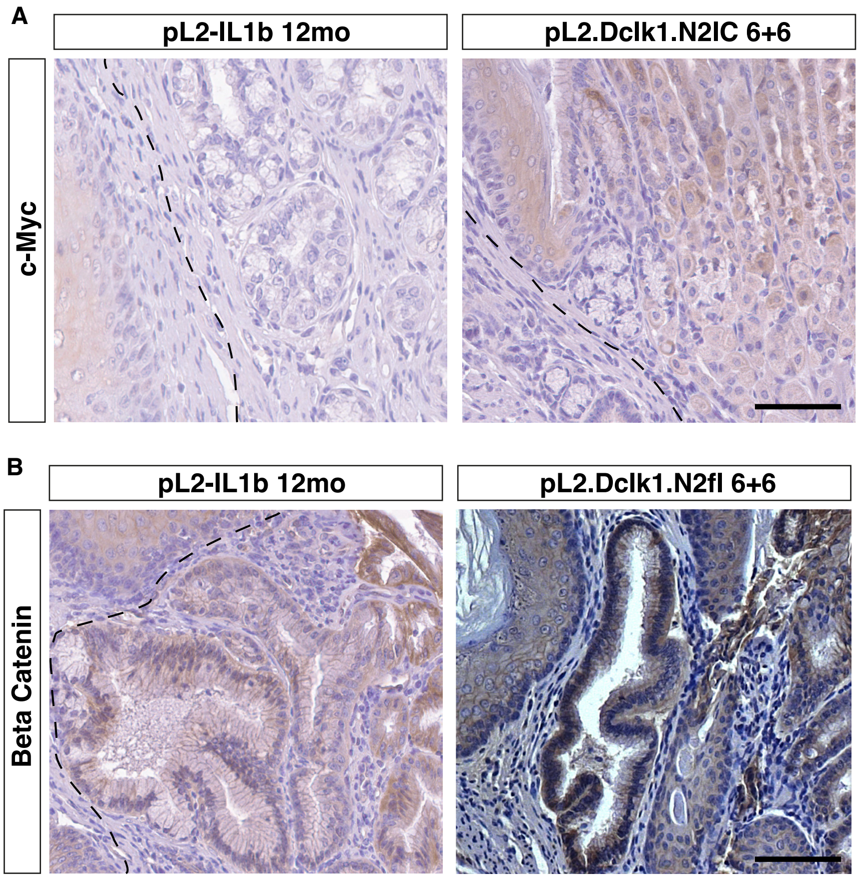

Supplement: Supplementary file 2 — Supplementary Figures. [file 41598_2021_84011_MOESM2_ESM.pdf]
